# Prevalence and associated factors of protein- energy wasting among patients with chronic kidney disease at Mulago hospital, Kampala-Uganda: a cross-sectional study

**DOI:** 10.1186/s12882-018-0920-7

**Published:** 2018-06-14

**Authors:** Lydia Namuyimbwa, Collins Atuheire, Joel Okullo, Robert Kalyesubula

**Affiliations:** 1 Department of Physiology, College of Health Sciences, School of Biomedical sciences, Makerere University, P.O Box 7076, Kampala, Uganda; 2 Department of Biosecurity, Ecosystems and Veterinary Public Health, College of Veterinary Medicine, Animal Resources and Biosecurity (BEP), Makerere University, Kampala, Uganda; 3 Department of Public Health, School of Allied Health Sciences, Kampala International University, Bushenyi, Kampala, Uganda

**Keywords:** Protein energy wasting, Chronic kidney disease, Anthropometric measurements

## Abstract

**Background:**

Chronic kidney disease (CKD) is global health concern and priority. It is the 12th leading cause of death worldwide. Protein Energy Wasting occurs in 20–25% of patients with chronic kidney disease and can lead to a high morbidity and mortality rate. We determined the prevalence of protein energy wasting and factors associated among patients with chronic kidney disease at Mulago National Referral Hospital, Kampala, Uganda.

**Methods:**

We conducted a cross-sectional study recruiting 182 (89 non-CKD patients and 93 CKD patients) consecutively from the outpatient clinic and wards on New Mulago Hospital complex. We took anthropometric measurements including heights, weights, Triceps skin fold (TSF), Mid- Upper Arm circumference (MUAC), Body Mass Index (BMI) and Mid-arm muscle circumference (MAMC). Serum albumin levels and lipid profile levels were also obtained. Following consent of study participants, Data was collected using questionnaires and analyzed using STATA 14.1. Percentages, frequencies, means, medians, standard deviation and interquartile range were used to summarise data. Crude and adjusted binary logistic regression was performed to assess unadjusted and adjusted effect measures of protein energy wasting due to several factors. Stratification by CKD status was performed during the analysis to minimize confounding.

**Results:**

The median age for CKD patients was 39 years compared to 27 years for non-CKD participants (*p* < 0.001). The prevalence of protein energy wasting (PEW) was 68.6% in this study with 47.3 and 21.3% among CKD and non-CKD participants respectively. Factors which were associated with PEW included CKD age between 18 and 24, being single, catholic religion, CKD stage 4, Hb < 11.5 g/dl and LDL > 160 mg/dl.

**Conclusion:**

Protein energy Wasting is prevalent among patients with chronic kidney disease and clinicians should routinely screen for it during patient care.

**Electronic supplementary material:**

The online version of this article (10.1186/s12882-018-0920-7) contains supplementary material, which is available to authorized users.

## Background

Chronic kidney disease (CKD) has become a global health concern worldwide. Its incidence is growing by around 8% annually making it the 12th leading cause of death globally. The prevalence of CKD in the general population in the United States of America(USA) is 14% and diabetes and hypertension are the leading causes of CKD [1]. The speculated increase in the burden of diabetes and cardiovascular disease in Africa to 130% by 2020 will lead to arise in the number of people suffering from CKD [2]. Prevalence of CKD in Uganda has been reported to be 15.2% and the risk factors for CKD include being female, age above 30 years and a high social economic class [3]. The Uganda Ministry of Health ministerial policy statement 2014/2015 allocated 0.01% of the total Ministry of Health budget to Non Communicable Diseases (NCDs) which is 3% of the total departmental budget. This is however supplemented by a five year grant from the World Diabetes Federation (WDF) which expires at the end of 2017. The total budget for NCDs is 27,000 USD per annum however this funding was increased by 25% of the departmental budget [4]. This shows that the burden of NCDs is increasing in Uganda and this has caused financial implications since it has put constraints on the Uganda National budget. Protein energy wasting (PEW) is common among patients with chronic kidney disease and it worsens as the glomerular filtration rate detoriates. The prevalence of PEW has been reported to be 20–25% in early to moderate CKD and increases as CKD progresses [5]. It can lead to increased number of hospitalization, increased susceptibility to infections and poor patient outcome [6]. PEW is a strong and independent predictor of death and quality of life in patients with kidney disease as well as graft loss in kidney transplant recipients. The international society of renal nutrition and metabolism (ISRNM) panel recommends use of four major categories for the diagnosis of PEW which include; serum chemistry, body mass, muscle mass and dietary intake. However diagnosis of PEW can be made if three characteristics are present(low serum levels of albumin, reduced body mass and reduced muscle mass) [7]. Chronic kidney disease being prevalent in Uganda (15.2%), it is important to study the nutrition status of CKD patients so that PEW can be diagnosed early, managed and prevented by clinicians during patient care. We hypothesized that there is a difference is in the nutrition status among CKD patients and participants without CKD. This is largely driven by the restrictive diets that are often recommended for CKD patients along with the metabolic alterations and inflammation that predispose them to wasting. Protein energy wasting (PEW) among patients with CKD at Mulago Hospital and Uganda at large has not been characterized. This creates a gap in the nutritional management of these patients due to lack of baseline data. We determined the prevalence of PEW and associated factors among patients with chronic kidney disease at Mulago hospital, using a cross sectional study.

## Methods

### Design and setting of the study

This was a cross sectional study involving patients with CKD aged 18 years and above and participants without CKD. The study was conducted at MulagoNational Refferal Hospital renal outpatients clinic.

### Study participants

This research was conducted at Mulago National Referral and teaching hospital. It is one of the three National referral hospitals in the country in addition to Mbarara and Butabika. Mulago National Referral Hospital is the largest hospital in Uganda and the teaching hospital of the Makerere University College of Health Sciences. It also serves as the general hospital for the Kampala metropolitan. It is one of the two national referral hospitals in Uganda, the other being Butabika National Referral hospital. It offers services in most medical and sub surgical specialties and the care system is public. The hospital has an official bed capacity of 1790 beds although it houses over 3000 patients. This hospital is representative of the Ugandan population because it is a national referral hospital where all cases are referred to for better management. At the time of conducting this study in 2013, Mulago Hospital was the only hospital with a dedicated clinic for patients with CKD in Uganda.

Patients with CKD were recruited from the renal out- patient clinic which is located at the fourth floor of Mulago Hospital. Participants without CKD were attendants of patients admitted on the wards in New Mulago hospital complex.

Patients with chronic kidney disease had a glomerular filtration rate of < 60 ml/min/1.73m^2^ for ≥3 months or any GFR with proteinuria, were aged 18 years and above and able to consent. Admitted patients and those on dialysis were excluded.

### Selection of participants

A systematic sampling procedure was used to enroll patients as they were registered in the renal unit out patients register. The expected number of participants to be enrolled on a particular day was divided by the number of patients who attended the clinic that day and systematic sampling method was used. The participants in the out patients’ clinic were met after their consultations with the physicians and participants were recruited on clinic days until the sample size was achieved.

Consent was sought first from the participants after adequate information was given about the study objectives, procedure and benefits of the study.

Participants without CKD were conveniently selected from the first to fourth floor of the New Mulago complex. Twenty two participants were conveniently selected from the four wards of the first three floors and twenty three were selected from the fourth floor.

### Study variables

The outcome variable, Protein Energy Wasting (PEW) was measured as participants who were having BMI < 18.5 kgm^− 2^ and serum albumin < 3.8 g/100 ml and Mid upper arm circumference (MUAC) > 10% in relation to 50th percentile which is 22.5 cm in our study population [8].

Chronic Kidney Disease (CKD) was obtained using CKD- EPI equation:GFR=141 * min(Scr/κ,1)^α^* max(Scr/κ, 1)^-1.209^ * 0.993^Age^ * 1.018 [if female] * 1.159 [if black]

Scr is serum creatinine (mg/dL), κ is 0.7 for females and 0.9 for males, α is − 0.329 for females and − 0.411 for males, min indicates the minimum of Scr/κ or 1, and max indicates the maximum of Scr/κ or 1.

eFGR stages were classed as follows: CKD [yes; < 60 ml/min/1.73m^2^; No: ≥ 60 ml/min/1.73m^2^]. Then staging was done as follows: stage 1: > 90 ml/min/1.73m^2^; stage2: 60–90 ml/min/1.73m^2^; stage3: 30–59 ml/min/1.73m^2^; stage4: 15–29 ml/min/1.73m^2^; stage5: < 15 ml/min/1.73m^2^ [9].

The serum total cholesterol, triglycerides, low density lipoproteins (LDL), high density lipoproteins (HDL), serum albumin and CRP were then analyzed using the COBAS INTEGRA 400 PLUS chemistry analyzer. Hemoglobin was analyzed using coulter autoloader machine according to the manufacturer’s standard operating procedures and quality assurance instructions. The urine protein was determined using the standard urine dipstick method following manufacturer’s instructions (UriCheck 10 ®).

Weight and height were measured for each patient with CKD and participant without CKD and body mass index (BMI) calculated. The mid-upper arm circumferences (MUAC), triceps skin fold thickness (TSF) were measured and the mid-arm muscle circumference (MAMC) calculated. The CKD patients and participants without CKD had fasted for at least eight hours and the following investigations were carried out: lipid profile levels, serum albumin levels, urine protein, hemoglobin levels and C-reactive protein (CRP). All investigations were carried out in the chemistry laboratory of Mulago hospital which is CAP certified.

### Statistical analysis

All analyses were done using STATA 14.2 (Statacorp 4905 Lakeway Drive, College station Texas 77,845 USA). Participants were stratified into CKD and non-CKD groups basing on eGFR and baseline comparison was done using z-test for proportions or Median test/Smirnov Kolmogrov test for non-normally distributed data across the two strata. (See details of the data set in Additional file 1). Prevalence of Protein Energy Wasting (PEW) was summarized using percentages. Statistical comparison of proportions PEW across CKD and non-CKD was done using 95% CIs. To Control for confounding of CKD status, stratified analysis was performed. Crude binary logistic regression and Adjusted binary logistic regression were carried out of potential predictors on Protein Energy Wasting (PEW). Crude and adjusted odds ratios were reported respectively for both strata. At analysis, effect modification (using chunk test) and confounding (at 10% cut-off) were done appropriately. Statistical significance was considered at α ≤ 0.05.

## Results

### Socio-demographic factors of study participants

There were 93 participants with chronic kidney disease (CKD) and 89 without (non- CKD). There were significant differences in some of the socio-demographic factors among CKD and non-CKD participants. There were more females (126/182) than males (56/182) in our study. More females (60%, 73/126) were non CKD participants whereas more males (71%, 40/56) were CKD patients.

The median age for CKD patients was 39 years compared to 27 years for non-CKD participant (*p* < 0.001) as shown in Table 1 at the end of the document.

**Table 1:** Socio-demographic characteristics for the study population

Variable	Non-CKD patients (*n* = 89)	CKD patients (*n* = 93)	*p*-value
median age in yrs. (IQR)	27 (23–31)	39 (30–51)	< 0.001
Gender n (%)			
Male	16 (17.98)	40 (43.01)	0.01
Female	73 (82.02)	53 (57.0)	0.01
Education n(%)			
None	9 (10.2)	11 (11.8)	0.88
Primary	34 (38.2)	38 (40.7)	0.81
Secondary	31 (34.8)	29 (31.2)	0.75
high school	5 (5.6)	5 (5.4)	0.99
Tertiary	10 (11.4)	10 (10.8)	0.97
Religion n (%)			
Protestant	25 (28.1)	27 (29.0)	0.96
Catholic	32 (36.0)	37 (39.8)	0.81
Muslim	19 (21.4)	15 (16.1)	0.76
Pentecostal	1 (1.1)	2 (2.2)	0.95
Other	12 (13.5)	12 (12.9)	0.97
Occupation n(%)			
Unemployed	35 (39.3)	51 (54.8)	0.09
employed-salary earner	19 (21.4)	19 (20.4)	0.92
self-employed	35 (39.4)	23 (24.7)	0.11
Marital status n (%)			
Married	14 (15.9)	17 (18.3)	0.89
Cohabiting	42 (47.7)	30 (32.3)	0.34
Separated	1 (1.1)	4 (4.3)	0.85
Widowed	1 (1.1)	5 (5.4)	0.80
Single	30 (34.1)	37 (39.8)	0.73

### Anthropometric and biochemical parameters of study population

The major causes of CKD include; hypertension (48%), HIV (10.8%), Urological diseases (10.8%) and Diabetes mellitus (9.7%).

The median BMI was significantly lower (23.8 kg/m^2^) among patients with CKD compared to non CKD participants (25 kg/m^2^) with lower and upper quartile as 22 and 26.4 kg/m^2^ respectively.

The median serum albumin was significantly lower (39.9 mg/dl) among CKD patients compared to non-CKD participants (41.5 mg/dl) with lower and upper quartile as 31.7 and 43.4 mg/dl respectively. Median Cholesterol level (4.91 mg/dl) and LDL (3.48 mg/dl) for CKD patients were significantly higher than those for non-CKD participants (3.95 mg/dl) and (3.48 mg/dl) (*p* = 0.001 and *p* = < 0.001 respectively).

Patients with CKD had a higher median creatinine level, as high as 1.8 mg/dl compared to 0.7 mg/dl for non-CKD participants, *p* < 0.001. Similarly the median CRP among CKD patients was as high as 4.48 mg/dl with lower and upper quartiles as 1.96 and 11.06 mg/dl respectively, much higher than that among non-CKD participants(1.53), *p* = 0.001.

Median Haemoglobin level was significantly lower among CKD patients compared to non-CKD patients, 12 g/dl with lower quartile and upper quartile being 10.3 g/dl and 13.9 g/dl respectively as shown in Table 2.

**Table 2:** Anthropometric and biochemical parameters of the study population

Variable	Non CKD Participants	CKD Patients	*P* value
	*N* = 89	*N* = 93	
Mean MUAC(SD)	26.2 (3.1)	25.6 (3.3)	0.20
Median BMI in Kg/ m^2^ (IQR)	25 (22.5–27.0)	23.8 (22.0–26.4)	0.04
Median serum albumin (IQR)	41.5 (39.9–44.1)	39.9 (31.7–43.4)	0.001
Median creatinine in mg/dl(IQR)	0.67 (0.57–0.79)	1.81 (1.09–7.08)	< 0.001
Median total cholesterol in mmol/L(IQR)	3.95 (3.31–4.80)	4.91 (3.94–6.08)	0.001
Median HDL in mmol/L (IQR)	1.27 (1.01–1.54)	1.04 (0.79–1.34)	0.05
Median LDL in mmol/L (IQR)	3.48 (2.4–3.25)	3.48 (2.4–4.45)	< 0.001
Median Hb in g/dl (IQR)	13.3 (12.6–14.4)	12.2 (10.3–14.0)	0.008
Median CRP in mg/dl(IQR)	1.53 (0.65–3.51)	4.48 (1.96–11.06)	0.001

*BMI* Body Mass Index, *HDL* High Density Lipoproteins, *LDL* Low Density Lipoproteins, *Hb* Hemoglobin, *CRP* C-Reactive Protein, *MUAC* Mid Upper Arm Circumference

### Protein energy wasting among CKD patients

Prevalence of PEW among CKD patients was 44(47.3, 95%CI 37.2–57.5) as compared to 19(21.3, 95%CI 12.8–29.9.) among the non-CKD participants.

### Factors associated with protein energy wasting among CKD patients attending Mulago hospital, Uganda

Among CKD patients; those participants aged 18–24 years were 5 times more likely to develop PEW compared to those over 34 years [cOR = 4.9; 95%CI 1.2–19.5]. Participants who were single were 7.7 times more likely to develop PEW compared to those that were married [cOR = 7.7; 95%CI 1.9–31.5]. Catholics were 2.6 times more likely to develop PEW compared to participants who were of Anglican Faith [aOR = 3.4; 95%CI 1.01–11.3]. Participants who were salary earners were 70% less likely of developing PEW compared to those that were unemployed [cOR = 0.3; 95%CI 0.1–0.9].

HIV was positively associated with PEW, with about 3 times more risk of PEW among HIV positive as compared to those that were HIV negative [cOR = 2.9; 95%CI 0.7–12.0], however, the association was not statistically significant.

Patients with hemoglobin< 11.5 g/dl were approximately 3 times more likely to develop PEW compared to those with at least 11.50 g/dl [aOR = 2.85; 95%CI 1.0–8.4]. CKD patients with higher LDL, (> 160 mg/dl) were 2.9 times more likely to develop PEW compared to those with LDL of at most 160 mg/dl [cOR = 2.92; 95%CI 1.2–7.2].The stage of CKD was associated with PEW, especially CKD patients with 4th stage were 6.4 times more likely to develop PEW compared to stage 1 CKD as shown in Table 3 at the end of the document.

**Table 3:** Binary and Multiple logistic regression of factors associated with PEW among CKD study participants

	Protein energy Wasting (PEW)		Crude Logistic regression			Adjusted Logistic regression		
Variable n(%†)			cOR	95%CI	*p*-value	aOR	95%CI	*p*-value
For CKD patients (*N* = 93)	negative (*n* = 49)	positive (*n* = 44)						
CKD stages								
1st	11 (64.7)	6 (35.3)	1.00					
2nd	10 (76.9)	3 (23.1)	0.55	0.11–2.80	0.47			
3rd	8 (38.1)	13 (61.9)	2.98	0.79–11.25	0.11			
4th	2 (22.2)	7 (77.8)	6.42	1.00–41.2	0.05			
5th	18 (54.5)	15 (45.5)	1.53	0.46–5.11	0.49			
age in yrs								
> 34	35 (59.3)	24 (40.7)	1.00					
25–34	11 (52.4)	10 (47.6)	1.33	0.49–3.61	0.58			
18–24	3 (23.1)	10 (76.9)	4.86	1.21–19.53	0.026			
Gender								
Males	20 (50.0)	20 (50.0)	1.00			1.00		
Females	29 (54.7)	24 (45.3)	0.83	0.36–1.89	0.65			
Religion								
Protestant	18 (66.7)	9 (33.3)	1.00			1.00		
Catholic	16 (43.2)	21 (56.8)	2.63	0.94–7.36	0.07	3.38	1.01–11.27	0.048
Muslim	9 (60.0)	6 (40.0)	1.33	0.36–4.93	0.67	2.83	0.60–13.33	0.19
Pentecostal	1 (50.0)	1 (50.0)	2.00	0.11–35.81	0.64	2.80	0.05–148.9	0.61
Education								
None	7 (63.6)	4 (36.4)	1.00					
Primary	21 (55.3)	17 (44.7)	1.42	0.35–5.66	0.62			
Secondary	13 (44.8)	16 (55.2)	2.15	0.52–9.00	0.29			
high school	2 (40.0)	3 (60.0)	2.63	0.30–23.0	0.38			
Tertiary	6 (60.0)	4 (40.0)	1.17	0.20–6.80	0.86			
Occupation								
Unemployed	23 (45.1)	28 (54.9)	1.00					
employed-salary earner	14 (73.7)	5 (26.3)	0.29	0.09–0.94	0.038			
self-employed	12 (52.2)	11 (47.8)	0.75	0.28–2.02	0.57			
marital status								
Married	14 (82.3)	3 (17.7)	1.00			1.00		
Cohabiting	17 (56.7)	13 (43.3)	3.6	0.84–15.08	0.08	2.37	0.45–12.40	0.31
Separated	3 (75.0)	1 (25.0)	1.56	0.12–20.6	0.34	1.8	0.09–34.54	0.70
Widowed	1 (20.0)	4 (80.0)	18.67	1.50–232.29	0.023	8.26	0.46–147.81	0.15
Single	14 (37.8)	23 (62.2)	7.67	1.87–31.49	0.005	6.20	1.24–31.15	0.027
Diabetes								
No	44 (52.4)	40 (47.6)	1.00					
Yes	5 (55.6)	4 (44.4)	0.88	0.22–3.51	0.86			
Hypertension								
No	25 (52.1)	23 (47.9)	1.00					
Yes	24 (53.3)	21 (46.7)	0.95	0.42–2.15	0.90			
urologic disease								
No	45 (54.2)	58 (45.8)	1.00					
Yes	4 (40.0)	6 (60.0)	1.78	0.47–6.76	0.40			
Hb in g/dl								
11.50–18.0	35 (62.5)	21 (37.5)	1.00			1.00		
< 11.5	14 (37.8)	23 (62.2)	3.0	1.27–7.08	0.012	2.85	1.00–8.44	0.05
CRP in mg/dl								
≤ 2.99	22 (57.9)	16 (42.1)	1.00					
> 2.99	27 (49.1)	28 (50.9)	1.43	0.62–3.28	0.40			
HIV								
No	46 (55.4)	37 (44.6)	1.00					
Yes	3 (30.0)	7 (70.0)	2.9	0.70–12.0	0.14			
LDL in mg/dl								
≤ 160 mg/dl	37 (61.7)	23 (38.3)	1.00			1.00		
> 160 mg/dl	11 (35.5)	20 (64.5)	2.92	1.19–7.20	0.02	3.06	0.95–9.92	0.062
HDL in mg/dl								
< 40 mg/dl	24 (52.2)	22 (47.8)	1.00					
≥ 400 mg/dl	25 (53.2)	22 (46.8)	0.96	0.43–2.17	0.92			

*HDL* High Density Lipoproteins, *LDL* Low Density Lipoproteins, *Hb* Hemoglobin, *CRP* C-Reactive Protein

## Discussion

The results obtained from this study showed that Protein -energy wasting (PEW) was present in 47.3% (44/93) of the CKD patients compared to 21.3% (19/89) of the participants without CKD. This figure of 47.3% in this study is of significance to Uganda being a third world country. This however lies within the reported 20 to 50% in the developed world [10]. This figure also agrees with findings in India where the prevalence of PEW among chronic kidney patients was 42.7% [11]. Several studies have reported presence of PEW among dialysis patients than among other CKD groups; however this study highlights the presence of PEW in the non dialysis kidney disease patients. It is important for clinicians to monitor routinely PEW in chronic kidney disease patients during patient care since PEW is a strong predictor of morbidity and mortality among this population [12]. These finding also have consequences as regards the management of patients with CKD in Uganda. PEW increases progression to ESRD as well as poor patient outcome. With the fewer dialysis centers available in Uganda and the high cost for dialysis, this can cause financial constraints on the affected families and the country as well since part of the dialysis cost is covered by the Ugandan government. Early diagnosis and treatment of PEW among this population will delay progression to End stage Renal Disease so the government costs on dialysis will reduce [13].

The median LDL was significantly high among the patients with CKD as compared to participants without CKD [3.48(2.40–4.45) vs 3.48(2.4–3.25)] *P* = < 0.001.These finding compare with what was found in Jos university teaching hospital, Nigeria where the mean LDL was also significantly higher in the study group compared to the controls (2.90+ 1.56 mmol/L vs 1.87 + 1.00 mmol/L) [14]. These findings show that the LDL levels in this study were borderline high and since 48% of the CKD patients have hypertension as factor which would accelerate the development of coronary artery disease, maintaining their levels below 2.5 mmol/L would be protective against coronary artery disease.

The median total cholesterol was higher among the patients with CKD as compared to the participants without CKD [4.91(3.94–6.08) vs 3.95(3.31–4.80)] *P* = 0.001. These findings differ from Binyam Mamuye’s findings where the mean total cholesterol was similar in both the kidney patients (166.8 ± 47.5 mg/dl) and the control group (166.3 ± 48.4 mg/dl) *P* = 0.400 [15]. Findings from this study show that the total cholesterol is still within the normal ranges. These patients may probably be on cholesterol lowering drugs for which this was not taken under consideration in this study which might have been one of our limitations .

The median CRP among CKD patients was as high as 4.48 mg/dl with lower and upper quartiles as 1.96 and 11.06 mg/dl respectively, much higher than that among non-CKD patients, *p* = 0.001 which was similar to what was reported in Nigeria. The increased levels of CRP in this study population indicate the inflammatory response that usually occurs with chronic kidney disease. However it has also been reported that CRP is a valuable predictor of cardiovascular risk in CKD and it also plays a role in atherosclerosis. Levels> 3 mg/dl correspond to a high risk for cardiovascular events, therefore patients in this study are at a high risk for cardiovascular events and therefore they should be monitored and interventions done in time to prevent cardiovascular events [16].

### Factors associated with PEW

These finding of PEW being associated with age group 18–24 may probably be as a result of these young adults not being well familiar with the diet they are supposed to feed on as CKD patients. By their tender age, they are most likely to be psychologically affected by the disease more than any other age group. In Uganda it’s around this age that people finish school and start to think about starting their own families, this makes this age group to become more worried about their future and life span hence being at risk of developing PEW. Being single is a factor that can predispose to depression especially if someone has a chronic illness; therefore supportive care in form of counseling is usually helpful to single patients. Uganda is a third world country with limited resources especially health workers. Scarcity of health workers who are in position to support patients psychologically creates a gap on how patients cope with the disease positively and they end up being depressed. This may alter their appetite leading to the development of PEW. A patient having CKD and HIV is more likely to develop PEW because both illnesses can lead to wasting. The prevalence of HIV in Uganda has increased to 7.3% and the existence of both conditions together worsens PEW. LDL levels > 160 can predispose a patient to cardiovascular disease and the two conditions occurring together can increases the chances of developing PEW. Catholic region doesn’t forbid its followers from taking alcohol so patients with CKD and are Catholics are more likely to take alcohol which can impair their judgment and appetite leading to wasting. It is of importance that religious leaders preach against alcoholism especially emphasis being put on individuals with chronic illnesses. Patients in stage 4 are more likely to develop protein energy wasting since at this stage the levels of inflammation is so much and the increased levels of proinflammatory cytokines lead to anorexia and hence insufficient protein and energy intake. This agrees with what was reported by Csaba where by PEW is more common in the later stages of CKD and that stages 4 and 5 are more likely to be accompanied by worsening protein energy status [5].

Our study had several strengths; we were able to recruit a comparative arm to highlight the prevalence of PEW among CKD and non CKD participants. We undertook a comprehensive assessment of the anthropometric measurements for all the participants providing a comprehensive physical and biomedical assessment of wasting among our study participants.

However, our study had a few limitations, particularly on the area of dietary assessment. The dietary assessment is important in enabling us to assess the caloric intake of participants and may also help one to establish the cardiovascular risks associated with high caloric intakes and their contribution to CKD. This association could not be ascertained in this study. There are few studies which have used the ISRNM Criterion to diagnose PEW and this has brought a gap in the comparison of findings from this study with findings from other settings where the same criterion has been used.

## Conclusion

This study showed that PEW is common among patients with chronic kidney disease. Clinicians should routinely monitor and screen patients for PEW during patient care so as to reduce morbidity and mortality rates as well as improve patient outcome. Emphasis should not only be put on dialysis patients but also on the other stages of CKD since occurrence of PEW in these groups has been documented. This study was designed to determine the prevalence and associated factors of PEW among patients with chronic kidney disease at Mulago hospital. Further studies analyzing the contributions of dietary interventions and whether they can lead to improved patient outcome as regards to management of PEW are needed to develop policies and measures to prevent wasting.

## Additional file

Additional file 1: Questionnaire. (ZIP 22 kb)

## Abbreviations

BMI: Body Mass Index
CKD: Chronic Kidney Disease
CRP: C- Reactive Protein
eGFR: Estimated Glomerular Filtration Rate
ESRD: End Stage Renal Disease
ESRD: End Stage Renal Disease
HDL: High Density Lipoproteins
ISRNM: International Society of Renal Nutrition and Metabolism
LDL: Low Density Lipoproteins
MAMC: Mid Arm Muscle Circumference
MUAC: Mid-Upper Arm Circumference
NCDs: Non communicable Diseases
PEW: Protein Energy Wasting
SGA: Subjective Global Assessment
TSF: Triceps skin fold
WDF: World diabetes federation
